# Deep learning-based semantic vessel graph extraction for intracranial aneurysm rupture risk management

**DOI:** 10.1007/s11548-022-02818-6

**Published:** 2023-01-10

**Authors:** Annika Niemann, Daniel Behme, Naomi Larsen, Bernhard Preim, Sylvia Saalfeld

**Affiliations:** 1 Department of Simulation and Graphics, Otto-von-Guericke University, Magdeburg, Germany; 2 STIMULATE Research Campus, Magdeburg, Germany; 3 University Clinic for Neuroradiology, Otto von Guericke University, Magdeburg, Germany; 4 Department of Radiology and Neuroradiology, University Medical Center Schleswig-Holstein (UKSH), Kiel, Germany

**Keywords:** Intracranial aneurysm, Geometric deep learning, Aneurysm rupture risk

## Abstract

**Purpose:**

Intracranial aneurysms are vascular deformations in the brain which are complicated to treat. In clinical routines, the risk assessment of intracranial aneurysm rupture is simplified and might be unreliable, especially for patients with multiple aneurysms. Clinical research proposed more advanced analysis of intracranial aneurysm, but requires many complex preprocessing steps. Advanced tools for automatic aneurysm analysis are needed to transfer current research into clinical routine.

**Methods:**

We propose a pipeline for intracranial aneurysm analysis using deep learning-based mesh segmentation, automatic centerline and outlet detection and automatic generation of a semantic vessel graph. We use the semantic vessel graph for morphological analysis and an automatic rupture state classification.

**Results:**

The deep learning-based mesh segmentation can be successfully applied to aneurysm surface meshes. With the subsequent semantic graph extraction, additional morphological parameters can be extracted that take the whole vascular domain into account. The vessels near ruptured aneurysms had a slightly higher average torsion and curvature compared to vessels near unruptured aneurysms. The 3D surface models can be further employed for rupture state classification which achieves an accuracy of 83.3%.

**Conclusion:**

The presented pipeline addresses several aspects of current research and can be used for aneurysm analysis with minimal user effort. The semantic graph representation with automatic separation of the aneurysm from the parent vessel is advantageous for morphological and hemodynamical parameter extraction and has great potential for deep learning-based rupture state classification.

## Introduction

Intracranial aneurysms are deformations of the vessel wall. Due to their location, the treatment of intracranial aneurysms is challenging and highly patient-specific. The necessity of treatment depends among other factors on the likelihood of an aneurysm rupture. The rupture risk assessment of intracranial aneurysm is a time-consuming task consisting of several steps. In clinical practice, medical history, sex and age are important factors for treatment decisions. Several other approaches exist in clinical research; for example, morphological parameters are used for rupture prediction. Also, evaluation of hemodynamics based on blood flow simulations yields important factors for rupture risk assessment. Both analysis methods require segmentation of the aneurysm in the image and generation of a 3d surface model. For reliable and consistent results, an automatic analysis is desired.

In clinical routine, intracranial aneurysms are often evaluated based on the PHASES [1] and the UIATS score [2]. The PHASES score consists of population, aneurysm size, hypertension, earlier subarachnoid hemorrhage, patient age and site of the aneurysm. The UIATS score is more complex and includes three categories: patient, aneurysm and treatment. The patient category includes age, risk factors (for example previous subarachnoid hemorrhage, smoking, alcohol abuse), symptoms and comorbid disease. In the aneurysm category, maximum diameter, irregularity, size ratio, aspect ratio and location are used [3]. These scores are simple to use in clinical routines. But management of unruptured intracranial aneurysms is still controversial, and the information captured by the score is limited. Especially for patients with multiple aneurysms, these scores are not very well-suited [4]. In research, current models for aneurysm rupture probability include age, gender, aneurysm location and a large number of variables derived from aneurysm geometry and hemodynamic simulation [4, 5]. To extend the information used in clinical routines, these should be extracted with minimal effort for physicians.

In this paper, we propose a semantic graph segmentation and centerline extraction for improved aneurysm rupture risk analysis. Based on semiautomatic image segmentation, we adopt a deep learning-based approach for the mesh segmentation. From the semantic segmentation of the mesh, a detailed abstract graph representation of the aneurysm is automatically generated. This is combined with automatic centerline calculation and outlet detection. Based on this information several approaches for rupture prediction can be carried out like shape parameter calculation, scripted hemodynamic simulations and aneurysm classification.

## Related work

In this section, related concepts for aneurysm analysis including aneurysm rupture risk assessment are described.

### Image segmentation

The MATCH study [6] comprised a challenge where 26 international computational fluid dynamics (CFD) research groups participated. It revealed that the most popular segmentation algorithms for intracranial aneurysms in 3d DSA datasets are threshold-based and level-set methods followed by region growing and watershed algorithms. After an initial segmentation is performed, further, often manual, preprocessing like smoothing, cropping and artifact removal is carried out. Deviations in the segmentation can lead to a difference of up to 25% in morphological parameters [6].

**Fig. 1:**
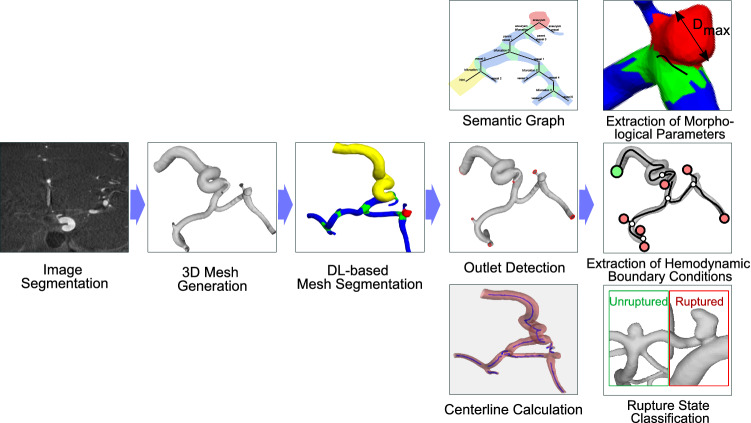
Overview of our intracranial aneurysm processing and analysis pipeline. The DL-based mesh segmentation allows for extraction of the semantic graph and automatic outlet and centerline detection. This favors the extraction of morphological parameters, the approximation of hemodynamic boundary conditions and the rupture state classification for rupture risk prediction

### Mesh segmentation

For extraction of morphological parameters or for carrying out a hemodynamic simulation, a 3d surface model of the aneurysm and its parent vessels is generated. A separation of the aneurysm from the surrounding vasculature is necessary to extract different parameters, and this separation is often done manually or semi-automatically [7]. Yang et al. [8] compared several deep learning algorithms for segmentation and separation of aneurysm and parent vessel. They found that segmentation on point clouds was superior to methods based on voxels. In general, the segmentation suffered for aneurysms which were small compared to the parent vessel. Points close to the vessel were sometimes misclassified by point-based methods. This problem did not occur with MeshCNN [9] which performs deep learning on meshes.

MeshCNN [9] uses mesh pooling for deep learning segmentation and classification. The pooling operation consists of an edge collapse operation converting five edges into two. The segmentation net consists of pooling and unpooling layers to restore the original mesh resolution. An advanced version of MeshCNN is MedMeshCNN^1^ [10]. It allows deep learning on larger meshes and training on data with class imbalance, which is crucial for clinical aneurysm models. Like MeshCNN, MeshNet [11] applies deep learning to surface meshes. In contrast to the edge-based MeshCNN, MeshNet proposes deep learning for mesh classification based on faces. From each face, the center point, vectors to the three vertices of the face, the unit normal vector and the indices of the connected faces are used as features. With convolution blocks, the features are summarized in a global feature vector which is used for classification. In contrast to a deep learning mesh segmentation methods, Kaick et al. [12] presented a mesh segmentation which first splits the mesh in weakly convex parts and then combines parts with similar geometric properties.

### Centerline calculation

Antiga et al. [13] proposed a vessel centerline calculation using Voronoi diagrams. Each point of the Voronoi diagram is assigned to a maximal inscribed sphere. Centerlines between given points minimize the integral of the radius of the maximal inscribed spheres along the path.

### Semantic graph

Antiga et al. [14] also developed an algorithm for graph representation of vessels. Models of carotid bifurcations were successfully decomposed into separate branches. Based on the centerline and the maximum inscribed sphere radius, the models were divided into branches. Branches connect at splitting lines. They tested their algorithm on idealized models of branching vessels. Based on their algorithm Chnafa et al. [15] generated a reduced-order model for estimation of outflow rates with less computational resources compared to 3D simulations. Their model consists of nodes and edges with information like length, equivalent radius and vectors at the extremities.

Saalfeld et al. [16] used a vessel graph extracted based on the centerline for outflow-splitting. The graph included the inlet vessel, outlet vessels and bifurcations. The aneurysm itself was not visible in the graph representation.

### Rupture risk

In a review of risk factors [17], 59 morphological and 55 hemodynamic parameters were collected. Factors with a high level of evidence for aneurysm rupture were irregular aneurysm shape, larger aspect ratio (aneurysm height divided by neck diameter), larger size ratio (aneurysm height divided by parent vessel diameter) and higher bottleneck factor (aneurysm width divided by neck diameter). The parent artery diameter was found to have inconsistent evidence regarding the aneurysm rupture risk [17].

Detmer et al. [5] developed an aneurysm rupture probability model and tested 25 morphological and 22 hemodynamic parameters. Besides gender, age and aneurysm location, 12 geometric and 11 hemodynamic parameters were used in the final model. Another factor connected to aneurysm rupture is the aneurysm location. Forget et al. [18] reported aneurysms at the anterior communicating artery were prone to rupture. The aneurysm location is also used in UIATS and PHASES scores [3].

Hemodynamic simulations can be used for assessment of aneurysm rupture risk as well as for investigation aneurysm evolution mechanisms [19]. For their study, Cebral et al. [19] classified the surface mesh into aneurysm, near vessel and far vessel. The distinction in near and far vessel was based on the distance to the ostium, and vessel surface with more than 1 cm distance to the ostium was defined as far.

Invantsits et al. [20] used machine learning on morphological features, gray-level radiomics features and features based on graphs to predict aneurysm rupture. They constructed a directed graph from the carotid or vertebral artery to the aneurysm. The length and volume of this path and the number of bifurcations were used as features. For up to three bifurcations, a binary encoding depicts whether the aneurysm is located in the larger vessel sub-tree. The inclusion of these features improved the rupture risk prediction.

**Fig. 2:**
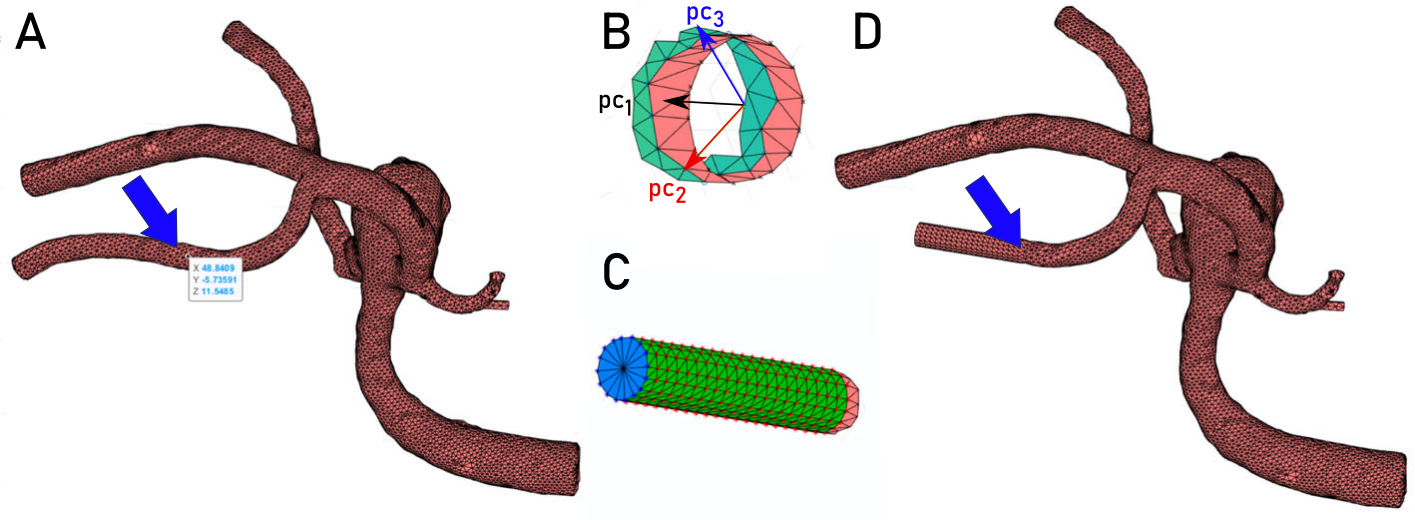
Illustration of the mesh cutting. **A** The user clicks on the surface (see arrows). **B** Mesh points within a predefined distance are used to determine a local center. The orientation is determined via principal component analysis, and the first three principal components ($pc_1$ - $pc_3$) are depicted. **C** The mesh is extruded five times of the local vessel diameter (approximated from the local center and the distance to the mesh points. **D** The resulting mesh is generated by stitching the extruded part and replacing the anatomically bended vessel part (see arrow)

## Material and methods

Diagnosis and rupture risk assessment for intracranial aneurysms consists of several steps (see Fig. 1). From a segmented image, a surface mesh of the aneurysm is generated. The focus of this work is the processing of the mesh for rupture risk prediction. The first step is the semantic segmentation of the mesh in several parts. This semantic segmentation is further refined by analyzing the part position in relation to each other and generating a semantic graph representation with detailed labels for each part. With automatic outlet and centerline detection, the mesh can be used for several tasks related to rupture risk prediction.

### Medical image data

For the presented method, we used a total of 84 aneurysm surface models. The datasets were acquired at the University Hospital of Magdeburg and at the University Hospital Schleswig-Holstein by analyzing 3D digital subtraction angiography data as part of the necessary clinical work-up performed on an Artis Q (Siemens Healthineers, Forchheim, Germany) or an 3T MR scanner (Achieva, Philips Healthcare, Best, The Netherlands). We furthermore include surface models of the Aneurisk study [21]. Thus, we account for possible variations due to imaging modalities and scanning protocols.

### Aneurysm surface model extraction

For the extraction of aneurysm surface models, the medical image data sets were segmented via threshold-based segmentation. Segmentation masks were converted into 3D surface meshes via Marching Cubes. Blending artifacts were removed and vessel were cut perpendicularly More information regarding the segmentation process is provided in previous work [7, 22]. Segmentation and surface mesh results are checked with clinical experts to account for an anatomically plausible vessel representation.

### Mesh cutting

For the mesh cutting, a software prototype was developed in MATLAB 2020a (The MathWorks, Inc., www.mathworks.com). This step ensures that stable flow profiles can be developed during hemodynamic simulations. These simulations require the usage of perpendicular outlets. The user selects a vertex of the mesh at the inlet or outlets and a neighborhood size (e.g., 3 mm). Next, all mesh points within the neighborhood of the selected point are used for a principal component analysis in order to approximate the local direction and center at the part of the vessel, see Fig. 2. Finally, the mesh is cut and perpendicularly extruded to the local direction of the vessel part. As a rule of thumb, we apply an extrusion five times the vessel diameter, but the user can change this parameter in case that vessel segments would intersect each other.

**Fig. 3:**
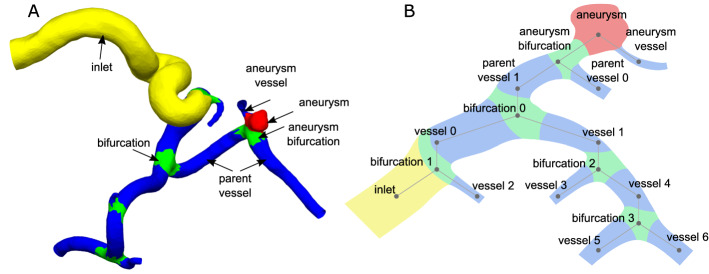
**A** Example of mesh segmentation of intracranial aneurysm; the difference between aneurysm vessel, parent vessel and other vessel is not made by the segmentation but derived from the semantic graph representation. **B** Semantic graph representation based on the example provided on the left

### Mesh segmentation and aneurysm graph extraction

Based on the requirements for subsequent analysis and hemodynamic simulation, the surface mesh of the aneurysm and the surrounding vessels are segmented into four classes. The first class (yellow) collects edges of the inlet vessel, which is characterized by an enlarged vessel diameter. The aneurysm itself, colored in red, makes up the second class. Bifurcations are illustrated by the green areas and include the crossover sections between different vessels or the aneurysm. All remaining vessels, highlighted in blue, are collected in the fourth class (see Fig. 3a). For performing this segmentation, MedMeshCNN is used [10]. A weighted loss function was chosen due to the imbalanced classes of the aneurysm meshes, i.e., the classes contained significantly smaller amounts of aneurysm and bifurcation classes. The 3D models were downsampled to an edge count of 19,200 edges, which provides the best trade-off between the precision of the input and computational costs. The performance metric was based on the intersection over Union (IoU) which quantifies the overlap between the ground truth classes and the predicted classed. More details regarding MedMeshCNN as well as annotation of the training data is provided in [10]. We trained our net with 60 aneurysm meshes and tested it on 24 aneurysms. The selection of this datasets was based on the internally available datasets with known rupture state.

Based on the part segmentation, a semantic graph representation of the aneurysm and the vessels is generated. The segmentation of the parts works on edges and returns a label for each edge. The segmentation does not further distinguish between two different vessels. This distinction is added after the segmentation. Each part assigned a unique numeric label, where the hundreds digit represents the label given by mesh segmentation. Lower digits are used to generate unique numeric identifiers for each segment. For visualization of the graph (Fig. 3b), the digits are mapped back to their respective labels. For each vertex, the labels of the corresponding edges are analyzed. In case different edge labels at the same vertex occur, these indicate a connection between the two parts. A graph reflecting the part connections is build.

The next step is analyzing the graph to gain more information about the relation between the different parts (see Fig. 3b). Based on the number of bifurcations between a vessel and the aneurysm, each vessel is assigned a level. Vessels directly at the aneurysm are labeled as aneurysm vessel. The bifurcation underneath the aneurysm is labeled as the aneurysm bifurcation. Parent vessels are vessels directly connected to the aneurysm bifurcation. This process automatically refines the segmentation labels and provides helpful information for further processing.

### Centerline and outlets

Outlets can be defined in the following way. For meshes with open ends, the outlets can be detected by searching edges which only belong to one triangle. For closed meshes, the outlets can be detected by analyzing the angle between neighboring triangles (recall Section “Mesh segmentation and aneurysm graph extraction”). Angles close to 90 degree might indicate an outlet. A group of vertices with suitable degrees between triangles which additionally builds a circular, planar shape is identified as outlet.

The centerline of the aneurysms is calculated using vmtk [23]. This algorithm is prone to missing some branches of complex aneurysm models. To solve this, the centerline for each segment is determined individually. The seed points are automatically set based on an automatic outlet detection.

## Aneurysm analysis

After the mesh is segmented into parts and information about the parts is collected in the semantic graph and centerline as well as outlets are calculated, these can be used for various applications. The currently most common used approaches for rupture risk prediction, extraction of morphological parameters and analysis of hemodynamic simulations, benefit from the presented aneurysm processing. While several approaches for parent vessel reconstruction exist, this segmentation offers the possibility to solely measure the parent vessel without relaying on interpolated and approximated data. A new promising approach is deep learning mesh classification to distinguish ruptured and unruptured aneurysm.

### Morphological analysis

Our method provides two advantages for analyzing intracranial aneurysms. First, the separation from aneurysm and parent vessel is carried out, thus, the aneurysm neck curve can be identified and established morphological parameters can be automatically extracted. Second, the semantic graph representation itself can be used for morphological analysis.

Morphological parameters like maximum aneurysm diameter or aneurysm height to neck size ratio are important predictors for aneurysm rupture risk [5, 24]. For an automatic extraction, the definition of an aneurysm neck, i.e., the separation of the aneurysm from the parent vessel is necessary [7]. This step can be automatically carried out with our presented vessel graph representation. In addition, the vessel graph allows a precise calculation of the parent vessel diameter. The area beneath the aneurysm is identified as the aneurysm bifurcation. As the aneurysm could change the diameter at that part of the parent vessel, this section is excluded for the parent vessel diameter calculation. The start and end of the parent vessel are defined by the bifurcations. This allows a uniformly parent vessel evaluation and reliable comparison between parent vessels of different aneurysms. As parent vessel diameter either the average diameter of the parent vessel or the maximum diameter of the parent vessel can be measured.

In previous studies, the aneurysm location was an important factor in accessing aneurysm rupture. Therefore, they are included in the most common clinical scores, like PHASES score [25] and UIATS score [2]. Both assign a rupture risk based on different attributes including categories for the location. This opens up the questions why aneurysms at specific locations are more likely to rupture. The aneurysm location contains information about the local surrounding structures, for example the diameter and curvature of the vessels leading to the aneurysm. These information are extracted by using the vessel graph presentation. To assess the potential of the semantic vessel graph for rupture risk prediction, we extracted and analyzed the number of vessels per level, the average curvature and average torsion of vessels and the vessel length. For the analysis, we used 23 ruptured and 30 unruptured aneurysm surface meshes.

### Preprocessing for hemodynamic simulations

Hemodynamic simulation can provide valuable information for aneurysm rupture risk assessment. At the moment, the surface mesh is often manually preprocessed for the simulation, for example cutting and inlet and outlet definition. Therefore, results may vary and the process is time-consuming. The presented segmentation and semantic graph can be a step toward scripted, automatic simulations.

For hemodynamic simulations, inlets and outlets are defined. The position of these are crucial for the simulation results. To keep the conditions constant over several aneurysm and receive comparable results, a constant inlet and outlet definition is required. With the aneurysm graph segmentation, information about the vessel level are extracted. These can be used to decide on the included vessels around the aneurysms, for example only the parent vessel or all vessels up to a certain level. As the bifurcation areas are known, it is easily avoidable to cut those areas, as they might produce misleading outlets.

The distinction into near and far vessels by Cebral et al. [19] was based on the distance to the ostium. The presented segmentation indirectly includes the segmentation of the ostium as the edges between the aneurysm and the aneurysm bifurcation. Therefore, the provided segmentation can be used to automatically generate the classification described by Cebral et al. [19]. A major disadvantage of the solely distance based method is the risk to get hemodynamic measures at bifurcation instead of vessels. Taking the semantic segmentation into account this can prevent this issue.

### Classification

Instead of morphological parameters, we try to directly predict aneurysm rupture on surface meshes. Using 32 meshes for training and 6 for testing, deep learning classification of aneurysm surface meshes was performed. The classification was done with MeshNet. Three different data sets were generated based on the part segmentation and the semantic graph: just the aneurysm itself, aneurysm and parent vessel, and the whole aneurysm.

## Results

In this section, the results of the mesh segmentation and the semantic graph generation are presented and compared to other methods. Additionally, the results of the aneurysm analysis with morphological parameters and mesh classification are presented.

### Mesh segmentation

The test accuracy was 83.3%. The segmentation accuracy of the parts differs. The highest intersection over union was achieved for vessels (74.8%) and aneurysm (71.4%). Slightly less correct segmented was the inlet (69.8%), and the most problematic part to segment was the bifurcations (37.0%).

Once the training is completed, the aneurysm segmentation can be performed automatically. Automatic segmentation for aneurysm meshes focuses on separating aneurysm and parent vessel. This segmentation is more advanced and allows a complex and meaningful segmentation of the mesh.

**Fig. 4:**
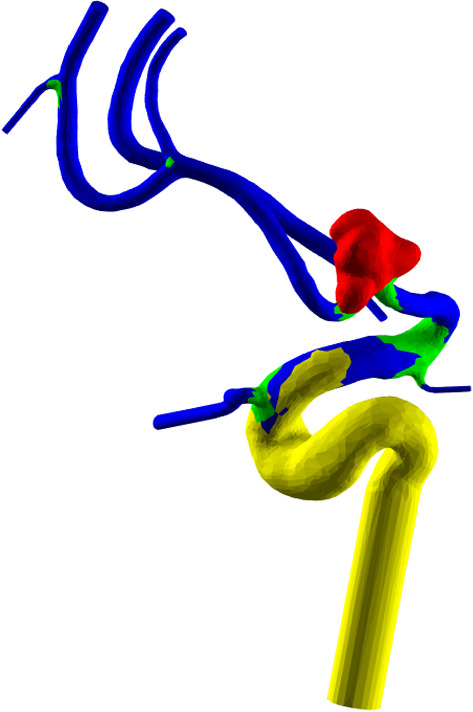
Result of the deep learning mesh segmentation. Aneurysm is colored red, bifurcations are colored green and the inlet is colored yellow. The remaining vessels are colored blue

**Fig. 5:**
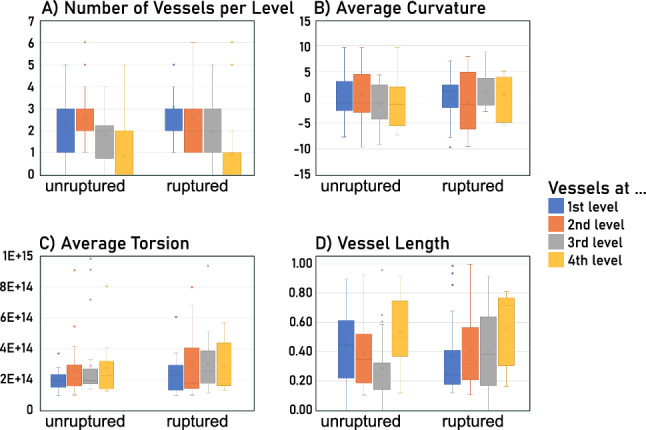
Comparison of attributes of the semantic vessel graph for unruptured and ruptured aneurysms. The parameters for the first four levels (colored blue, orange, grey and yellow) are depicted. **A** Number of vessels per level. **B** Average curvature per vessel level. **C** Average Torsion per vessel level. **D** Average vessel length per vessel level

### Results of morphological analysis

In contrast to focusing on established scores like PHASES or UIATS, we compared the semantic vessel graph and its attributes for ruptured and unruptured aneurysms.

For the first four vessel levels, the number of vessels at this level, the average vessel length at this level and average, minimum, maximum and variance of radius, curvature and torsion are calculated (see Fig. 5). As most of the meshes used in this study only have vessels up to level four, only the first four levels were included for further analysis. The vessel levels correspond to the number of bifurcations between the vessel and the aneurysm (see color-coding in Fig. 5). A small surface mesh or a large mesh with few bifurcations will both result in a limited number of vessel levels. All parameters are extracted based on the semantic graph representation and the centerline. For the evaluation, 23 ruptured and 30 unruptured aneurysm surface meshes were used.

Figure 5A shows the number of vessels occurring at each vessel level. Ruptured aneurysms tend to have slightly more vessels at level two and three compared to unruptured aneurysms. The average torsion and curvature for all levels was slightly higher in ruptured aneurysms than in unruptured aneurysms (see Fig. 5B, C). The average vessel length of the first two levels was higher in unruptured aneurysms.

### Classification

Mesh classification for meshes of the aneurysm, aneurysm and parent vessel and aneurysm with surrounding vessel were performed. The highest accuracy was archived for the meshes consisting of aneurysm and parent vessel. In Table 1, the best results for each set are shown, with an overall best result of 83.3% test accuracy for using the whole vascular domain.

**Table 1:** Training and test accuracy for aneurysm classification using MeshNet

Data	Training	Test
Aneurysm	0.906	0.833
Parent	0.968	0.833
Whole	0.909	0.750

## Discussion

**Fig. 6:**
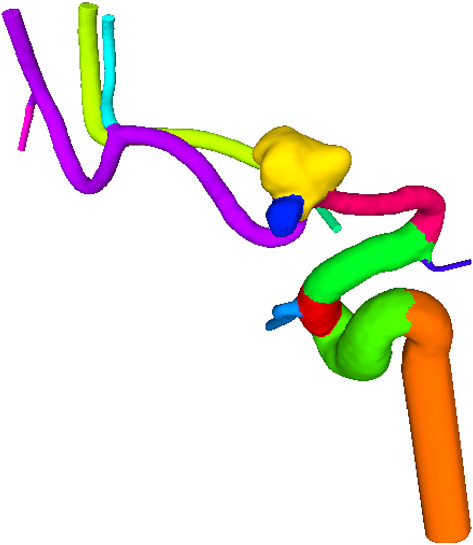
Results of the automatic part segmentation of Kaick et al. [12] for intracranial aneurysms. Note that the aneurysm is separated from its parent vessel, but the bleb (blue color) of the aneurysm (yellow) is segmented separately. The same surface mesh as shown in Fig. 4 is depicted

We presented a deep learning-based mesh segmentation for intracranial aneurysm analysis. Kaick et al. [12] presented an mesh segmentation based on convexity. For aneurysms without blebs, it is able to automatic segment the aneurysm and several vessel parts. However, these parts are missing the semantic interpretation offered by our deep learning segmentation in combination with our semantic vessel graph. Additionally, we can better capture the wide variation of aneurysm shapes, for example aneurysms with blebs. As shown in Fig. 6, their method extracts the bleb as additional class of the mesh classification, whereas our method is able to separate the aneurysm including the bleb.

A graph representation of aneurysms has been used for simulations. We compare our semantic graph with other aneurysm graphs. Chnafa et al. [15] introduced a graph representation of aneurysms for outflow rate estimation. The presented segmentation provides the necessary information for these abstraction. Unlike our aneurysm graph, their graph splits each bifurcation into several parts. We reduce this unwanted effects with our semantic graph. The determination of bifurcations allows for determination of flow splitting for hemodynamic simulations by defining the boundary conditions, as presented in previous work [16]. With the presented semantic graph representation and the extracted centerline, the bifurcations and corresponding radii can be automatically calculated and the extraction of boundary conditions can be speed up. Neither of the graph representations show the aneurysm itself. The semantic graph presented here does include the aneurysm.

Our semantic graph and following aneurysm analysis is based on deep learning mesh segmentation. While the experiments presented here show promising results, there are several limitations. An important and difficult to overcome challenge is the collection of sufficient data. At the moment, data augmentation techniques for meshes are largely limited. Three-dimensional deep learning is a very active research field. However, at the moment, it is rarely used in practical applications. Often, small meshes are used. Several algorithm specify additional expectations (for example, watertight non-manifold meshes for MeshCNN).

Preprocessing of the meshes was applied to decrease the variance in the number of faces. While Yang et al. [8] focused on the aneurysm and parent vessel, our segmentation covers a larger part of the vessels around the aneurysm. This increases the previous problem of segmentation of imbalanced classes. Additionally, we have four different classes instead of two.

In contrast to Antiga et al. [14], we explicitly define a bifurcation area. By splitting a mesh solely into vessel, the bifurcation area is added to several vessels. As the geometry changes at the bifurcation area, this leads to changes in the vessel diameter and geometry at the end. By excluding the bifurcation area from the vessel, we can calculate more reliable vessel parameters.

The parent vessel diameter is used to predict aneurysm rupture as a risk factor itself and in the calculation of the size ratio. Correct measurement of the diameter is necessary. Including the diameter at the neck, like Duan et al. [26], might lead to an overestimation of the parent vessel diameter due to the influence of the aneurysm. The challenge of correctly calculating the parent vessel diameter might contribute to the inconsistent results regarding the influence on the rupture risk [17]. By segmenting the aneurysm bifurcation, this area can be excluded for the diameter calculation.

The presented work builds the foundation for further research questions. As shown, several new options for automatic cutting of the vessels around the aneurysm arise from the part segmentation. These should be evaluated by hemodynamic simulation. In this work, the surrounding vessels as one contribution to rupture risk was introduced. To further explore why the aneurysm location is connected to the rupture risk, other location-specific properties, for example other surrounding structures, should be evaluated.

We presented a new segmentation for aneurysm meshes. The task of automatic segmentation was addressed with deep learning on triangle meshes. While showing promising results, an increased data set and further training is necessary to improve the segmentation. More segmentation following a similar concept are necessary to quantitatively compare segmentation results.

The semantic vessel graph provides additional information to the semantic segmentation. The labels of the segments are further refined by their position in relation to the other segments. This increases the usefulness of the semantic segmentation without making the deep learning segmentation too complex by adding more classes and increasing the problem of imbalanced classes.

First evaluation of morphological parameters of surrounding vessels show interesting results. However, these are limited by the data set size and the amount of vessels included in the aneurysm mesh. It would be helpful to analyze aneurysms where more surrounding vessels are preserved. The variation in the morphological parameters was large. For the higher vessel levels, less data were available, resulting in less reliable information about these.

Mesh classification with deep learning might be applied to predict the rupture risk of intracranial aneurysms. The classification task seems to benefit from the inclusion of the parent vessel. Further research has to evaluate the desired mesh size and classification algorithm for reliable mesh classification. When comparing our initial results for rupture status classification, we achieve promising results of 83.3% based on the mesh classification (recall Sect. 5.3). For comparison, classification based on established morphological parameters achieved an accuracy of 69% for intracranial aneurysms, with 79% for side wall and 68% for bifurcation aneurysms [27]. Since our database for mesh classification is rather small, these results must be validated with a larger database.

The described pipeline focuses on rupture prediction. Rupture risk is one of the main factors for treatment decisions. Another aspect are treatment risks. With inclusion of more information, for example patient age and overall health, the pipeline could be extended to include assessment of treatment risks. Combining both in a fully automatic pipeline could save time in aneurysm diagnosis.

## Conclusion

We present a new approach on intracranial aneurysm analysis. Using deep learning segmentation on surfaces meshes, a segmentation in several parts is presented. From this, a semantic graph representation of the parts is build. Outlets, centerline and morphological parameters are automatically detected, allowing for a script-based calculation of hemodynamic parameters. Further applications for this in aneurysm classification and simulation were discussed.

## Footnotes

1 https://github.com/LSnzd/MedMeshCNN.
